# Mental Health Disturbances and Related Problems in Italian University Medical Students from 2000 to 2020: An Integrative Review of Qualitative and Quantitative Studies

**DOI:** 10.3390/medicina57010011

**Published:** 2020-12-24

**Authors:** Gaia Sampogna, Giovanni Marcos Lovisi, Francesca Zinno, Valeria Del Vecchio, Mario Luciano, Érika Gonçalves Loureiro Sol, Roberto José Gervásio Unger, Antonio Ventriglio, Andrea Fiorillo

**Affiliations:** 1 Department of Psychiatry, University of Campania “L. Vanvitelli”, 80138 Naples, Italy; francesca.zinno@yahoo.it (F.Z.); valeria.delvecchio78@gmail.com (V.D.V.); mario.luciano@unicampania.it (M.L.); andrea.fiorillo@unicampania.it (A.F.); 2 Institute of Studies in Collective Health—Federal University of Rio de Janeiro, Rio de Janeiro 21941-901, Brazil; giovannimlovisi@gmail.com (G.M.L.); erika.gsol2@gmail.com (É.G.L.S.); roberto_unger@iesc.ufrj.br (R.J.G.U.); 3 Department of Clinical and Experimental Medicine, University of Foggia, 71100 Foggia, Italy; a.ventriglio@libero.it

**Keywords:** mental health problems, medical school, smoking, alcohol abuse, anxiety disorders, depressive disorders, suicidal behaviours

## Abstract

*Background and objectives*: The presence of mental health problems in the population of medical students in Italy has been evaluated in several cross-sectional studies, which have used different methodologies and study designs. However, a global overview of the prevalence of mental health problems in Italian medical students is not available, although this would be essential for promoting preventive strategies and supportive treatments. *Materials and Methods*: An integrative review aiming to describe the prevalence of mental health problems in Italian medical students has been performed. *Results*: The most relevant findings are the high prevalence of substance use, in particular alcohol and nicotine, and of depressive and anxiety disorders in Italian medical students. In particular, substance use ranges from 13 to 86%, which is higher compared to Italian students coming from other faculties. Italian medical students show a high rate of smoking and of depressive symptoms of about 20%. *Conclusions*: Our findings highlight the need to develop appropriate supportive interventions for the medical student population, which are rarely provided and implemented among the routine activities of Italian medical universities. A relevant aspect to be considered is the stigma and anticipated discrimination attached to mental disorders, which reduce the help-seeking process in medical students.

## 1. Introduction

Up to 35% of university students suffer from common mental disorders or related health problems [1]. The transition from high school to university is a critical period in terms of biological, psychological, and social development, with the creation of new relationships, a new identity and an increase in autonomy and responsibility [2]. Moreover, most mental illnesses arise and develop during late adolescence.

The admission into medical school and the period of graduation are very competitive and highly stressful. In fact, compared with their peers, students from medical school show higher rates of mental health problems, including depressive and anxiety symptoms [3], and are at higher risk of using illicit substances [4] or of developing full-blown mental disorders [5]. Students reporting these symptoms have reduced academic performances and a consequent poor quality of provided healthcare and increased medical errors [6]. Risk factors for the development of psychiatric symptoms in this student population include female sex, exposure to recent stressful life events, excessive use of smartphone and low quality of sleep [7].

Tobacco consumption and smoking dependence represent a rising problem for medical students. In fact, Mas et al. [8] found that medical students’ tobacco consumption increases during the years of training in medical schools. Moreover, tobacco use has been associated with the risk of developing depressive symptoms [9]. Another unhealthy lifestyle behaviour frequently adopted by medical students is the consumption of energy drinks, which are used for their stimulatory effect and for claims of giving benefits, such as physical endurance reaction, concentration, and reduced need for sleep. Smoking represents a risk factor for the use of energy drinks in university students [10,11,12]. Therefore, the use of energy drink, smoking habits and alcohol consumption are considered “proxy” measures of the global mental health of medical students.

Despite medical students frequently have mental health problems, their levels of help-seeking are quite low [13]. It may be that their access to specialist care is delayed by the presence of stigma still attached to mental disorders, as recently documented by the American Psychiatric Association that more than 50% of medical students meeting the diagnostic criteria for a mental disorder are reluctant to seek professional help because of fear of exposure to stigma [14]. Moreover, after graduation, the fear of stigma and of financial and professional consequences are a significant barrier to seeking help among doctors [15,16,17]. In Italy, the levels of stigma attached to mental disorders are still quite high, particularly in specific socio-cultural contexts impacting negatively on the help-seeking. In particular, a survey carried out in the general population indicates that depression is usually seen as a reaction to significant life events. Moreover, the levels of stigma towards depression are quite high, as highlighted by the fact that participants report that it is embarrassing to report depressive symptoms to general practitioners and there are several concerns regarding the use of antidepressants [18].

The prevalence of mental health problems in Italian medical students has been evaluated in several studies, adopting different methodologies and study designs. However, a global overview of the prevalence of mental health problems in Italian medical students is not available, although this would be essential for promoting preventive strategies and supportive treatments. Therefore, we decided to perform an integrative review of qualitative and quantitative studies aiming to describe the prevalence of mental health problems in medical students in Italy, where we expect that the levels of mental health problems in medical students are higher compared to other countries [19], which could be due to the high levels of stigma, the prestigious role of medical doctors [20] and the lack of a structured organization of psychological support during medical training [21].

## 2. Materials and Methods

This review was performed in five stages: definition of the problem, literature search, data evaluation, data analysis and presentation of findings [22]. Our goal was to define the prevalence rates of mental health problems such as depression, anxiety or suicidal behavior or abuse/dependence of drugs among Italian medical students.

The following search terms, “students”, “medical”, “mental health”, “Italy”, “mental disorders”, “depression”, “anxiety”, “suicidal ideation”, “suicide attempted”, “suicide”, “alcoholism (alcohol and drug use)”, “street drugs (abuse, dependence, binge)”, “prevalence” and “risk factors” were entered in ERIC, MEDLINE, PsycARTICLES, PsycINFO, SCOPUS and PUBMED (Table 1). Terms and databases were combined using the Boolean search technique, which consists of a logical information retrieval system (two or more terms combined to make search more restrictive or detailed).

Three authors from Italy (GS, FZ and VG) and three authors from Brazil (EGLS, RJGU and GML) independently searched papers, which had been published between 1 January 2000 and 30 June 2020. Authors (GS, FZ, VG, EGLS, RJGU and GML) searched the same databases. The selected articles were saved in an Excel shared document.

Qualitative and quantitative studies on the prevalence of mental health problems (depression, anxiety, suicidal behavior and drug abuse/dependence) among Italian medical students, which had been published in peer-reviewed English or Italian journals (with abstracts written in English), have been included in the review. When studies included mixed student populations (i.e., medical and non-medical students) and it was not possible to extrapolate data only on medical students, these studies were excluded.

### 2.1. Selection of Study and Data Extraction

**Figure 1:**
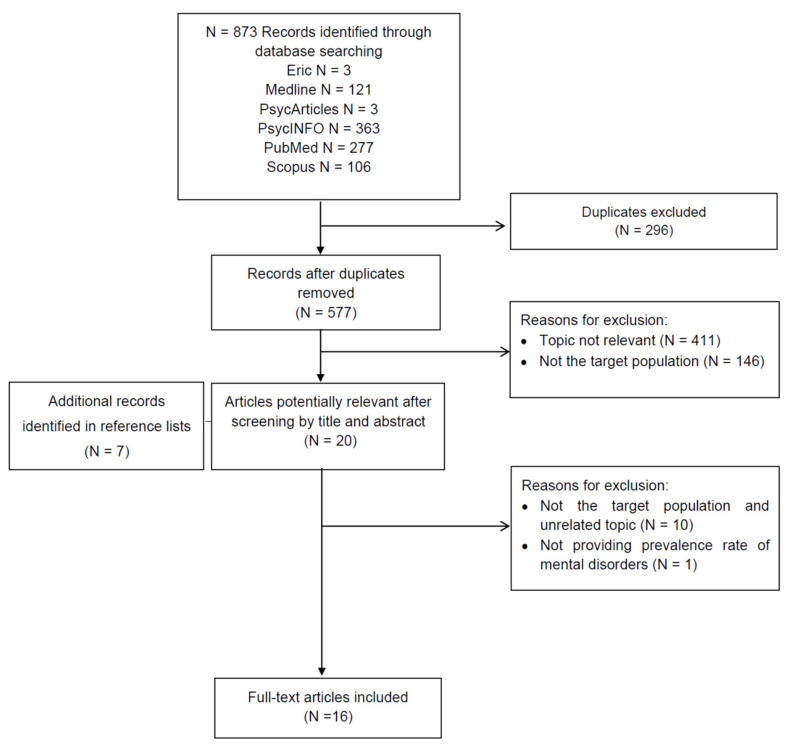
PRISMA flowchart.

Two members of the review team (one in Italy and one in Brazil) independently screened titles, removed duplicated papers, evaluated eligible studies, and performed data extraction. Any discrepancies in the study selection were resolved through discussion with a senior expert reviewer. Preferred Reporting Items for Systematic Reviews and Meta-Analysis (PRISMA) was used to demonstrate the selection of study [23] (Figure 1). Data from each study included: study design, sample size, prevalence of depression, anxiety or suicidal behavior or drug abuse/dependence among Italian medical students, mean age, sex, main aims, data collection, risk factors, main conclusions and GRADE (Grading of Recommendations, Assessment, Development and Evaluations) criteria [24].

### 2.2. Quality Assessment According to the GRADE Criteria

The quality of quantitative studies was evaluated according to the GRADE criteria by two authors from Italy (GS and FZ) and two from Brazil (RJGU and GML), independently. Four levels of quality are identified, from “high” to “very low”. The quality of evidence is considered “high” when the study is a randomized controlled trial; and “low” in the case of observational studies. The attribution of a lower level is based on the presence of limitations, bias, inconsistencies, indirectness, and impreciseness in the study.

The authors independently assessed the studies against these criteria and a discrepancy rate of about 10% was found, which was solved through online discussion.

## 3. Results

According to the search strategy, 873 records were identified. Based on title and abstract’s review, 296 were duplicates and were excluded. For the remaining 577 papers, the main reasons for exclusion were “topic not relevant” (N = 411) and “not the target population” (N = 146). The remaining 20 articles were retained, and seven additional eligible papers were identified by searching the references’ lists of the papers. Therefore, sixteen studies have been included in the review (Figure 1). In the majority of cases [25,26,27,28,29,30,31,32,33,34,35], the articles’ title was related to the use of cigarette, energy drink or alcohol. In 12 out 16 studies, the main aim was the evaluation of the consumption rate among Italian medical students and their knowledge and attitudes about substance use. Four studies [34,36,37,38] investigated the prevalence of alcohol, depressive symptoms and suicidal behavior, and evaluated the factors associated with these syndromes (Table 2).

The sample size of included studies ranged from 44 to 794 participants. Almost all studies adopted a cross-sectional design, while only three studies [29,32,35] used a multicenter cross-sectional design.

The prevalence rates were 15.3–31.4% for cigarette use, 22–75% for energy drink and 13–86% for alcohol use. The percentage of smokers was significantly higher in male than female students [26]. Moreover, male students were at higher risk of using multiple substances (drink, alcohol, nicotine and coffee) and of being heavy smokers compared to female students [33]. However, women showed a higher prevalence of depressive and anxiety symptoms [36].

On the global sample, the prevalence of depressive symptoms ranged between 2.5 and 21.4%, while severe suicidal thoughts were found in 17% of participants [37]. Depressive symptoms and deficit/hyperactivity symptoms were more likely to be present among students seeking counseling [38]. Students with severe suicidal behavior had higher rates of anxious and cyclothymic temperaments compared with non-severe suicidal group [36].

Only one study [38] with a small sample size (N = 44) found a higher prevalence rate of adaptation disorder (36%) and of psychotic disorder (14.3%), compared to dysthymia and panic disorder (7.1%).

The levels of knowledge regarding the risks of tobacco use, its epidemiological aspects and possible treatments were relatively low among Italian medical students.

Regarding data collection, thirteen studies [25,26,27,28,29,30,31,32,33,34,35,36,39] used self-reported instruments which had been administered during class time. In the remaining studies [37,38], data collection took place during a medical examination or at the counseling center. A 75–100% response rate was found for data collection during class time, while a lower response was found if the recruitment took place through email invitation.

### Summary of Design Quality

The GRADE criteria were adopted to evaluate the quality of included studies. The majority of the studies (N = 10, 62.5%) were of moderate quality, being cross-sectional studies and conducted in one or two cities only. The remaining six papers were rated of low quality due to the small sample size and the lack of limitations or to the risk of reported bias. Since the overall quality of the included studies is moderate, we recommend that multicenter longitudinal studies involving students attending different years of training in the medical schools should be carried out.

## 4. Discussion

To our knowledge, this is the first integrative review aiming to evaluate the prevalence rate of mental health problems in Italian medical students. While many healthcare professionals report high rates of burnout and poor mental health and wellbeing, it is likely that the student population is even more vulnerable to the development of mental health problems [40,41,42,43]. Several studies have reported that medical students have a poor mental health and a low access to adequate treatments [44,45,46,47,48,49,50,51]. Furthermore, the mean age of access to a medical school corresponds with the mean age of onset of many mental disorders. The highly competitive environment of university settings could represent a trigger factor for vulnerable students, thus precipitating the onset of any mental disorder [52]. It is likely that mental health can worsen when the medical school starts and can remain poor during the whole training period.

Based on the included studies, the most striking findings are the high prevalence of substance use, in particular alcohol and nicotine, and of depressive and anxiety disorders. In particular, substance use in Italian medical students is very high, ranging between 13–86%, which is higher compared to Italian students coming from other faculties. In a study by Jackson et al. [52] in the US, alcohol abuse and dependency among medical students was associated with higher levels of burn out. This finding can be due to the fact that expectations at medical schools are very high, since students are trained to become future scientists and health professionals.

Compared with other European students, Italian medical students show the second highest rate of smoking [53]. A systematic review by Niu [54] on a Chinese population of medical students found some improvements in the status of tobacco use, following an educational campaign against tobacco targeted to medical students. Moreover, while the prevalence of smoking is about 20% in the Italian general population, it is slightly higher (ranging between 15–31%) in Italian medical student population [55]. However, we found that the prevalence rates of smoking of Italian medical students is lower compared with those of Italian students from other university settings. This finding may be due to the increased knowledge on the detrimental effects of tobacco during medical training, which can contribute to reduce the consumption of nicotine along the years [56].

In line with previous studies carried out in Europe, the prevalence rate of depressive symptoms was about 20% in Italian medical students. However, Rotenstein et al. in their metanalytic study [3] found a wide variability of the prevalence of depression in medical students worldwide. We found an association between the presence of depressive symptoms and cyclothymic temperament, which highlights the importance of screening programmes in order to evaluate personality traits in students [57,58,59,60,61,62,63]. Furthermore, depressive symptoms are more frequently reported by female medical students compared with their male peers, thus confirming the higher prevalence of mood disorders in females. This finding needs a careful evaluation, considering that the number of female students starting a career in the medical field has doubled since 1980s. Therefore, it may be useful to develop preventive and supportive interventions for depressive disorders taking into account biological, social and psychological gender differences [64,65,66].

According to the reviewed studies, the prevalence of suicidal thoughts in Italian medical students is about 17%, and it is higher in males. This finding, which is consistent with previous evidence showing high rates of suicidal thoughts and behaviours in medical students [3,67,68,69], underlines the need to develop appropriate preventive strategies to tackle suicide risk in medical students [70,71].

The main limitations of included studies were the small sample sizes, the cross-sectional designs, and the use of self-reported instruments. In particular, when using self-reported instruments, the findings may be biased by the so-called “social desirability”. Moreover, these self-reported instruments should not be considered valid to formulate a diagnosis of a mental disorder, but they are rather screening tools. In order to evaluate the prevalence of mental health and related problems in medical students, there is the need to carry on longitudinal, multicenter, population-based studies with sound methodologies. Therefore, our findings must be interpreted cautiously, and further, longitudinal studies may be carried out in order to fill in this gap and obtain conclusive data.

The present integrative review has some limitations which should be acknowledged. Firstly, the decision to select and include only studies carried out in Italy makes our findings not representative of the global condition of mental health problems in medical students worldwide. However, this decision is due to the fact that the Italian context has some specific socio-cultural features which should be taken into consideration in order to develop ad hoc preventive interventions targeting medical students. Another limitation is due to the narrow time frame considered, but previous years have been covered by international systematic reviews [3].

## 5. Conclusions

Our findings confirm the high prevalence rates of depressive-anxiety symptoms and of substance abuse in the medical student population. Therefore, these data highlight the need to develop appropriate supportive interventions for this specific population [72,73], which are rarely provided and implemented among the routine activities of Italian medical universities. A relevant aspect to be considered is the stigma and anticipated discrimination attached to mental disorders, which reduce the help-seeking process in medical students. In particular, medical students feel concerned by being treated for a mental health disturbance, further delaying the first contact with psychiatric services [74,75,76,77,78]. On the other hand, treatment programmes for medical students with substance abuse should be developed, as well as training about smoking cessation [79,80].

## Figures and Tables

**Table 1:** Selection of the studies on mental health problems among Italian medical students.

Search Terms	Eric	Medline	Psyc Articles	Psyc INFO	PubMed	Scopus
Students, medical AND mental health AND Italy	0	77	2	105	62	29
Students, medical AND mental disorders AND Italy	0	18	0	77	76	22
Students, medical AND depression AND Italy	1	5	0	50	30	12
Students, medical AND anxiety AND Italy	1	6	0	50	27	16
Students, medical AND suicidal ideation AND Italy	0	0	0	10	6	2
Students, medical AND Suicide attempted AND Italy	0	0	0	8	4	1
Students, medical AND suicide AND Italy	1	3	0	23	22	8
Students, medical AND Alcoholism AND Italy	0	1	1	6	2	6
Students, medical AND street drugs AND Italy	0	2	0	3	2	3
Students, medical AND mental disorders AND Italy AND prevalence	0	8	0	18	34	3
Students, medical AND “mental disorders” AND Italy AND “risk factors”	0	1	0	13	12	4

**Table 2:** Selected characteristics of the studies on health-related behaviours among Italian medical students.

Author(s), Year	Study Design	Sample Size, Site(s)	Mean Age	Gender	Assessment Tool(s)	Main Aim(s)	Data Collection and Response Rate	Prevalence	Main Findings	Main Conclusions	Study Limitations	GRADE Criteria
Volpe et al. 2019	Cross-sectional	N = 360, Ancona, Foggia	Not reported	F: 58.33% M: 41.67%	Oldenburgh Burnout Inventory (OLBI); General Health Questionnaire (GHQ); CAGE (Cutdown/Annoyed/Guilty/Eye Opener)	To evaluate the general health and the levels of burnout	Online survey, invitation through institutional email to all university’s students. Response rate: Ancona 88.6%; Foggia: 11.4%	5%: anxiety disorder 2.5%: depression 0.28%: burnout syndrome 8.9%: alcohol related problems 33%: cognitive enhancers users	8.6% of participants reported mental health issues whilst at medical school, ranking as follows: Anxiety Disorders > Major Depression > Eating Disorders > ADHD > Burnout Syndrome.	The finding show that a certain percentage of Italian Medical students present psychological conditions with alcohol misuse and use of drugs to cope with their condition	Online survey; self-reporter instruments	MODERATE
Solano et al. 2019	Cross-sectional	N = 522, Genoa	23.6 ± 1.1 years	F:59% M: 41%	Suicide Opinion Questionnaire (SOQ); Coping Orientation to Problem Experienced (COPE); Temperament Evaluation of the Memphis (TEMPS-A)	To evaluate the presence of suicidal thoughts/behaviors, coping strategies, temperament and attitude towards suicidality in medical students	Collection during classrooms activities from medical students Response rate: 82.3%	17%: severe suicidal thought and behavior	The participants with poor coping strategies were more likely to be males, with poor academic achievement and less likely to have a parent working in medical or mental health fields.	The clinicians need to consider the complex interplay of clinical features present in medical students with severe suicidal behavior	Self-reported questionnaire; cross-sectional study	MODERATE
Pighi et al. 2018	Cross-sectional	N = 433, Modena and Reggio Emilia	22.4 years; Range: 18–48 years	F: 55.9% M: 44.1%	36-item ad-hoc validated questionnaire	To evaluate the prevalence of use of energy drinks and students’ attitudes	Collection during classrooms activities from medical students Response rate: 83.8%	74.7%: energy drinks users	Italian medical students used energy drinks and psychostimulant substances (coffee and caffeine) as cognitive enhancers very frequently	The use of psychostimulants is low and the majority of students are worried about academic performance.	Low generalizability of results; no use a standardized tool	MODERATE
Rapinesi et al. 2018	Case-control	N = 98 (N = 49, help-seekers; N = 49 control group), Sapienza-Rome	24.4 years (students seeking counseling); 21.7 years (non counseling controls)	Not reported	Beck Depression Inventory-II; Temperament and Character Inventory-Revised; Adult ADHD Self-Report Scale	To evaluate temperament, attention deficit/hyperactivity disorder traits and depressive symptomatology	Students attending 6-year of medical school and/or attending the counseling program Response rate: not available	17.1%: depressive symptoms in help-seeking group 10.1%: depressive symptoms in control group (*p* < 0.001) ADHD total score: 3.7 (help-seeking group) vs. 2.5 (control group) (*p* < 0.001)	Help-seeking students were more likely to have attention deficit/hyperactivity symptoms, scored higher on the Beck Depression Inventory-II and on the Temperament and Character Inventory-Revised	Medical students attending counseling center need to be carefully assessed for mental disorders	Small sample size	LOW RATE
Lamberti et al. 2017	Cross-sectional	N = 641, Naples	26.2 ± 5.5 years	F:59.1% M: 40.9%	Alcohol Use Disorders Identification Test (AUDIT -C)	To assess the prevalence of alcohol drinking in a large sample of students and residents	Collection during medical examinations in medical course students Response rate: 100%	85.5%: regular alcohol users 16.6%: regular smokers 91%: habitual coffee consumption	Over two-thirds of the students use alcohol regularly. Medical students use alcohol more frequently than residents. Over 90% of student use coffee regularly, one out five is active smoker and less than 60% perform physical activity	The results shown a need to assess alcohol use in healthcare professionals and to recognize risky behaviors in order to develop effective preventive interventions	Cross-sectional design, not representative sample	MODERATE
Armstrong et al. 2017	Multicenter cross-sectional	N = 527, Bologna	Not reported	M: 43.0%	International professional assessment of drinking and tobacco perceptions among young medical doctors	To evaluate the prevalence of medical students’ tobacco use, attitudes, clinical skills and tobacco-related curricula	Invitation through institutional email to all university’s students. Response rate: less than 40%	29.5%: smoking habits	Italian students were less likely to receive smoking cessation training compared to American students. Students reported to want to receive smoking cessation training	There is the need to include training on smoking cessation in medical course in order to reduce smoking among medical students, physicians and patients	Recruitment bias	MODERATE
Casuccio et al. 2015	Cross-sectional	N = 794, Palermo	21.9 ± 2.7 years; Range: 19–41	F: 52.5% M: 47.5%	Ad hoc questionnaire on consumption of energy drinks	To assess the levels of knowledge and attitudes related to energy drink consumption and the prevalence of side effects related to their use	Medical student of the 6- year course filling out the instruments during class time Response rate: 75%	22%: energy drinks users	Female students present high levels of somatization, obsessive-compulsive, depressive and anxiety symptoms. Mental disorders are more common in students using energy drinks regularly	There is an association between specific psychopathological characteristics and alcohol consumption	Small sample, cross-sectional design	MODERATE
Luca et al. 2015	Cross-sectional	N = 200, Catania	21.8 ± 3.1 years	F: 52.5% M: 47.5%	Alcohol Use Disorders Identification Test (AUDIT-C); Self-Report Symptom Inventory-Revised (SCL-90-R); General Symptomatic Index (GSI)	To assess the levels of alcohol consumption in relation with socio-demographic and psychopathological variables in a sample of medical students	Students attending the 6-year of medical schoolAdministration of the survey during classroom activities Response rate: 75%	27%: presence of mental health problems 13%: alcohol use disorder	Male students were most frequently regularly alcohol users 49% of participants consumed alcohol associated with energy drinks.	There is a great use of energy drinks among medical students, either alone or in combination with alcoholic beverages.	Cross-sectional design; self-reported questionnaires	LOW RATE
Lia et al. 2013	Cross-sectional	N = 44, Sapienza University-Rome	Not reported	Not reported	SCID-I -CV (The Structured Clinical Interview for DSM-IV Axis I Disorders-Structured clinical Interview)	To assess the prevalence of mental disorders among medical students attending a university counseling service	Students attending 6-year of medical school and/or attending the counseling program Response rate: not available	31.8%: axis I diagnosis 21.4%: depressive syndrome 35.7%: adaptation dis. 14.3%: psychotic dis. 7.1%: dysthymia 7.1%: anxiety 7.1%: panic attacks dis. 7.1%: bipolar dis. 7.1%: episode of mania	Services for psychological counseling targeting medical students should be present in Italian universities.	The prevalence of mental distress and mental disorder appears to be higher in medical students compared to the prevalence of the same problems in students attending other faculties	Small sample size, lack of comparison with other counseling services	VERY LOW RATE
Saulle et al. 2013	Multicenter cross-sectional	N = 730, Turin, Padua, Florence, Brescia, Ferrara, Varese Udine, Palermo, Salerno	Range: 19–29 years	F: 19.1% M: 22.4%	The Global Health Professions Student Survey (GHPSS)	To evaluate smoking prevalence, knowledge and attitudes among Italian medical students	Students attending third year of medical school Survey performed during classroom activities Response rate: 100%	20.4%: current smokers	87.7% believed that health professionals need to receive specific training on smoking cessation. 65% believed that health professionals had a role in giving advice or information about smoking cessation.	There is a need to provide medical students with smoking cessation training	Cross-sectional study design, self-reported data, recruitment bias (only third-year students)	MODERATE
Grassi et al. 2012	Cross-sectional	N = 439, Sapienza-Rome, Cattolica University-Rome, Udine, Verona	23.3 ± 3.0 years; Range: 20–55 years	F:61% M:49%	60-item ad-hoc validated questionnaire	To evaluate smoking behavior and to assess the levels of knowledge about smoking-related mortality, the harmful effects of cigarette smoking and the efficacy of counseling techniques	Self-administered questionnaires to students attending the course of pharmacology and toxicology Response rate: 40%	15.3%: current smokers 9.6%: previous smoker	The levels of knowledge on epidemiological aspects of tobacco use was low. A great proportion of students did not know how to provide counselling to smokers	Italian medical students do not receive adequate training on tobacco dependence	Small sample size	MODERATE
Gualano et al. 2012	Multicentre cross-sectional	N = 744, Rome, La Sapienza, Rome, Cattolica, Chieti, Turin, Palermo	Not reported	Not reported	The Global Health Professions Student Survey (GHPSS) questionnaire	To evaluate the prevalence of tobacco use, the levels of knowledge and attitudes about tobacco smoking cessation training	Self-administered questionnaires to all students interested Response rate: 98%	31.4%: current smokers	The great majority considered health professionals as role models for patients as well as they have a role in giving advice or information about smoking cessation	It should be useful to include smoking cessation training in the Italian medical course	Cross-sectional study, self-reported data, recruitment bias (only third year students)	MODERATE
La Torre et al. 2012	Multicenter cross-sectional	N = 655, Chieti, Palermo, Rome Sapienza, Rome Cattolica and Turin	21.34 years; Range: 20–44 years	F: 50.4% M: 49,6%	Global Tobacco Surveillance System form	To assess smoking prevalence; the levels of knowledge and attitudes toward smoking; the availability of tobacco cessation training	Second semester of the third year of medical school Response rate: 92%	29.3%: smoking habits	57.2% of participants believe that health professionals are role models for patients. 89.8% of them are aware of smoking cessation interventions.	The prevalence of smoking was higher among medical students than the general population. The training in smoking cessation techniques need to be provided to medical students.	Cross-sectional design, lack of representativeness	MODERATE
Lucenteforte et al. 2010	Cross-sectional	N = 194, Florence	Not reported	F: 65.8% M: 34.2%	Modified instrument of the World Health Organization about tobacco smoking for health caregivers	To assess the impact of university choice on smoking habits	First year students attending classroom lectures at the University of Florence Response rate: 100%	20.1%: smoking habits	32.5% of medical students had at least one parent who uses smoking	The medical students seem to be more conscious about the negative effects of smoking and tobacco-related diseases in comparison with other students.	Small sample size; not representative sample	LOW RATE
Oteri et al. 2007	Cross-sectional	N = 450, Messina	24.5 years; Range: 19–30 years	F: 58.7% M: 41.3%	Ad-hoc questionnaire about energy drink consumption	To evaluate the levels of knowledge related to the use of energy drinks (alone or in association with alcohol)	Anonymous survey Response rate: 90%	56.9%: use of energy drink 48.4%: use of energy drink associated with alcohol	The use of energy drinks and alcohol is frequent. The usage combined is associated to a higher risk of developing alcohol dependence.	The consumption of alcohol and energy drinks is high in medical student population.	Not reported	LOW RATE

## Data Availability

Data is contained within the article.

## References

[1] Auerbach R.P., Mortier P., Bruffaerts R., Alonso J., Benjet C., Cuijpers P., Demyttenaere K., Ebert D.D., Green J.G., Hasking P. (2018). WHO World Mental Health Surveys International College Student Project: Prevalence and distribution of mental disorders. J. Abnorm. Psychol..

[2] Taylor Z., Doane L., Eisenberg N. (2013). Transitioning from high school to college: Relations of social support, ego-resiliency, and maladjustment during emerging adulthood. Emerg. Adulthood.

[3] Rotenstein L.S., Ramos M.A., Torre M., Bradley Segal J., Peluso M.J., Guille C., Sen S., Mata D.A. (2016). Prevalence of depression, depressive symptoms, and suicidal ideation among medical students a systematic review and meta-analysis. JAMA J. Am. Med. Assoc..

[4] Mousa O.Y., Dhamoon M.S., Lander S., Dhamoon A.S. (2016). The MD Blues: Under-Recognized depression and anxiety in medical trainees. PLoS ONE.

[5] Moutinho I.L.D., Lucchetti A.L.G., da Ezequiel O.S., Lucchetti G. (2019). Mental health and quality of life of Brazilian medical students: Incidence, prevalence, and associated factors within two years of follow-up. Psychiatry Res..

[6] Agnafors S., Barmark M., Sydsjö G. (2020). Mental health and academic performance: A study on selection and causation effects from childhood to early adulthood. Soc. Psychiatry Psychiatr. Epidemiol..

[7] Lemola S., Perkinson-Gloor N., Brand S., Dewald-Kaufmann J., Grob A. (2015). Adolescents’ electronic media use at night, sleep disturbance, and depressive symptoms in the smartphone age. J. Youth Adolesc..

[8] Mas A., Nerín I., Barrueco M., Cordero J., Guillén D., Jiménez-Ruiz C., Sobradillo V. (2004). Smoking habits among sixth-year medical students in spain. Arch. Bronconeumol. Engl. Ed..

[9] Fluharty M., Taylor A.E., Grabski M., Munafò M. (2017). The Association of cigarette smoking with depression and anxiety: A systematic review. Nicotine Tob. Res..

[10] Azagba S., Langille D., Asbridge M. (2014). An emerging adolescent health risk: Caffeinated energy drink consumption patterns among high school students. Prev. Med..

[11] Miller K.E. (2008). Energy drinks, race, and problem behaviors among college students. J. Adolesc. Health.

[12] Pacheco J.P.G., Humes E.C. (2020). Personality traits, alcohol and cannabis use among medical students. Braz. J. Psychiatry.

[13] Gold J.A., Johnson B., Leydon G., Rohrbaugh R.M., Wilkins K.M. (2015). Mental health self-care in medical students: A comprehensive look at help-seeking. Acad. Psychiatry.

[14] Mehta S.S., Edwards M.L. (2018). Suffering in silence: Mental health stigma and physicians’ licensing fears. Am. J. Psychiatry Resid. J..

[15] Deb T., Lempp H., Bakolis I., Vince T., Waugh W., Henderson C., INDIGO READ Study Group (2019). Responding to experienced and anticipated discrimination (READ): Anti-stigma training for medical students towards patients with mental illness -study protocol for an international multisite non-randomised controlled study. BMC Med. Educ..

[16] Pingani L., Catellani S., Del Vecchio V., Sampogna G., Ellefson S.E., Rigatelli M., Fiorillo A., Evans-Lacko S., Corrigan P.W. (2016). Stigma in the context of schools: Analysis of the phenomenon of stigma in a population of university students. BMC Psychiatry.

[17] Thornicroft G., Bakolis I., Evans-Lacko S., Gronholm P.C., Henderson C., Kohrt B.A., Koschorke M., Milenova M., Semrau M., Votruba N. (2019). Key lessons learned from the INDIGO global network on mental health related stigma and discrimination. World Psychiatry.

[18] Munizza C., Argentero P., Coppo A., Tibaldi G., Di Giannantonio M., Picci R.L., Rucci P. (2013). Public beliefs and attitudes towards depression in Italy: A national survey. PLoS ONE.

[19] Volpe U., Luciano M., Palumbo C., Sampogna G., Del Vecchio V., Fiorillo A. (2014). Risk of burnout among early career mental health professionals. J. Psychiatr. Ment. Health Nurs..

[20] Sacchini D., Antico L. (2000). The professional autonomy of the medical doctor in Italy. Theor. Med. Bioeth..

[21] Volpe U., Ventriglio A., Bellomo A., Kadhum M., Lewis T., Molodynski A., Sampogna G., Fiorillo A. (2019). Mental health and wellbeing among Italian medical students: A descriptive study. Int. Rev. Psychiatry.

[22] Whittemore R., Knafl K. (2005). The integrative review: Updated methodology. J. Adv. Nurs..

[23] Moher D., Liberati A., Tetzlaff J., Altman D.G., Altman D., Antes G. (2009). Preferred reporting items for systematic reviews and meta-analyses: The PRISMA statement. PLoS Med..

[24] Ryan R., Hill S. (2016). How to GRADE the Quality of the Evidence. Cochrane Consumers and Communication Group. Version 3.0. http://cccrg.cochrane.org/author-resources.

[25] Melani A.S., Verponziani W., Boccoli E., Trianni G., Federici A., Amerini R., Vichi M., Sestini P. (2000). Tobacco smoking habits, attitudes and beliefs among nurse and medical students in Tuscany. Eur. J. Epidemiol..

[26] Oteri A., Salvo F., Caputi A.P., Calapai G. (2007). Intake of energy drinks in association with alcoholic beverages in a cohort of students of the school of medicine of the university of Messina. Alcohol. Clin. Exp. Res..

[27] Lucenteforte E., Vannacci A., Cipollini F., Gori A., Santini L., Franchi G., Terrone R., Ravaldi C., Mugelli A., Gensini G.F. (2010). Smoking habits among university students in Florence: Is a medical degree course the right choice?. Prev. Med..

[28] Gualano M.R., Siliquini R., Manzoli L., Firenze A., Cattaruzza M.S., Bert F., Renzi D., Romano N., Ricciardi W., Boccia A. (2012). Tobacco use prevalence, knowledge and attitudes, and tobacco cessation training among medical students: Results of a pilot study of Global Health Professions Students Survey (GHPSS) in Italy. J. Public Health.

[29] Saulle R., Bontempi C., Baldo V., Boccia G., Bonaccorsi G., Brusaferro S., Donato F., Firenze A., Gregorio P., Pelissero G. (2013). GHPSS Multicenter Italian Survey: Smoking Prevalence, Knowledge and Attitudes, and Tobacco Cessation Training among Third-Year Medical Students. Tumori J..

[30] Grassi M.C., Chiamulera C., Baraldo M., Culasso F., Ferketich A.K., Raupach T., Patrono C., Nencini P. (2012). Cigarette smoking knowledge and perceptions among students in four Italian medical schools. Nicotine Tob. Res..

[31] Lamberti M., Napolitano F., Napolitano P., Arnese A., Crispino V., Panariello G., Di Giuseppe G. (2017). Prevalence of alcohol use disorders among under- and post-graduate healthcare students in Italy. PLoS ONE.

[32] Armstrong G.W., Veronese G., George P.F., Montroni I., Ugolini G. (2017). Assessment of tobacco habits, attitudes, and education among medical students in the United States and Italy: A cross-sectional survey. J. Prev. Med. Public Health.

[33] Casuccio A., Bonanno V., Catalano R., Cracchiolo M., Giugno S., Sciuto V., Immordino P. (2015). Knowledge, attitudes, and practices on energy drink consumption and side effects in a cohort of medical students. J. Addict. Dis..

[34] Luca M., Ruta S., Signorelli M., Petralia A., Aguglia E. (2015). Psychological variables and alcohol consumption in a sample of students of medicine: Gender differences. Riv. Psichiatr..

[35] La Torre G., Kirch W., Bes-Rastrollo M., Ramos R.M., Czaplicki M., Gualano M.R., Thümmler K., Ricciardi W., Boccia A., GHPSS Collaborative Group (2012). Tobacco use among medical students in Europe: Results of a multicentre study using the Global Health Professions Student Survey. Public Health.

[36] Rapinesi C., Kotzalidis G.D., Casale A., Del Ferrone M., Vento A., Callovini G., Curto M., Ferracuti S., Sani G., Epompili M. (2018). Depressive symptoms, temperament/character, and attention deficit/hyperactivity disorder traits in medical students seeking counseling. Psychiatr. Danub..

[37] Solano P., Aguglia A., Caprino M., Conigliaro C., Giacomini G., Serafini G., Amore M. (2019). The personal experience of severe suicidal behaviour leads to negative attitudes towards self- and other’s suicidal thoughts and behaviours: A study of temperaments, coping strategies, and attitudes towards suicide among medical students. Psychiatry Res..

[38] Lia C., Lai E., Gallo V., Cavaggioni G. (2013). Characteristics of a population of medical students reported to the university student counseling service “fatti vivo”. Riv. Psichiatr..

[39] Pighi M., Pontoni G., Sinisi A., Ferrari S., Mattei G., Pingani L., Simoni E., Galeazzi G.M. (2018). Use and propensity to use substances as cognitive enhancers in Italian medical students. Brain Sci..

[40] Cohen K.A., Graham A.K., Lattie E.G. (2020). Aligning students and counseling centers on student mental health needs and treatment resources. J. Am. Coll. Health.

[41] Castaldelli-Maia J.M., Lewis T., Marques dos Santos N., Picon F., Kadhum M., Farrell S.M., Molodynski A., Ventriglio A. (2019). Stressors, psychological distress, and mental health problems amongst Brazilian medical students. Int. Rev. Psychiatry.

[42] Gross J.J., Uusberg H., Uusberg A. (2019). Mental illness and well-being: An affect regulation perspective. World Psychiatry.

[43] Puras D., Gooding P. (2019). Mental health and human rights in the 21st century. World Psychiatry.

[44] Castaldelli-Maia J.M., Martins S.S., Bhugra D., Machado M.P., De Andrade A.G., Alexandrino-Silva C., Baldassin S., Alves T.C.D.T.F. (2012). Does ragging play a role in medical student depression—Cause or effect?. J. Affect. Disord..

[45] Torales J., Kadhum M., Zárate G., Barrios I., González I., Farrell S.M., Ventriglio A., Arce A. (2019). Wellbeing and mental health among medical students in Paraguay. Int. Rev. Psychiatry.

[46] Cook A.F., Arora V.M., Rasinski K.A., Curlin F.A., Yoon J.D. (2014). The prevalence of medical student mistreatment and its association with burnout. Acad. Med..

[47] Milic M., Gazibara T., Pekmezovic T., Tepavcevic D.K., Maric G., Popovic A., Stevanovic J., Patil K.H., Levine H. (2020). Tobacco smoking and health-related quality of life among university students: Mediating effect of depression. PLoS ONE.

[48] Oquendo M.A., Bernstein C.A., Mayer L.E.S. (2019). A Key differential diagnosis for physicians—Major depression or burnout?. JAMA Psychiatry.

[49] Pacheco J.P., Giacomin H.T., Tam W.W., Ribeiro T.B., Arab C., Bezerra I.M., Pinasco G.C. (2017). Mental health problems among medical students in Brazil: A systematic review and meta-analysis. Braz. J. Psychiatry.

[50] Pedrelli P., Nyer M., Yeung A., Zulauf C., Wilens T. (2015). College students: Mental health problems and treatment considerations. Acad. Psychiatry.

[51] McGorry P., Trethowan J., Rickwood D. (2019). Creating headspace for integrated youth mental health care. World Psychiatry.

[52] Jackson E.R., Shanafelt T.D., Hasan O., Satele D.V., Dyrbye L.N. (2016). Burnout and alcohol abuse/dependence among U.S. medical students. Acad. Med..

[53] Panatto D., Amicizia D., Domnich A., Lai P.L., Cristina M.L., Signori A., Boccalini S., Sulaj K., Gasparini R. (2013). Tobacco smoking among students in an urban area in northern Italy. J. Prev. Med. Hyg..

[54] Niu L., Liu Y., Luo D., Xiao S. (2018). Current smoking behavior among medical students in Mainland China: A systematic review and meta-analysis. Asia Pac. J. Public Health.

[55] Lugo A., Zuccaro P., Pacifici R., Gorini G., Colombo P., La Vecchia C., Gallus S. (2017). Smoking in Italy in 2015–2016: Prevalence, trends, roll-your-own cigarettes, and attitudes towards incoming regulations. Tumori.

[56] Bassols A.M.S., Carneiro B.B., Guimarães G.C., Okabayashi L.M.S., Carvalho F.G., da Silva A.B., Cortes G.N., Rohde L.A.P., Eizirik C.L. (2015). Stress and coping in a sample of medical students in Brazil. Rev. Psiquiatr. Clin..

[57] Lesiewska N., Borkowska A., Junik R., Kamińska A., Pulkowska-Ulfig J., Tretyn A., Bieliński M. (2019). The association between affective temperament traits and dopamine genes in obese population. Int. J. Mol. Sci..

[58] Fico G., Luciano M., Sampogna G., Zinno F., Steardo L., Perugi G., Pompili M., Tortorella A., Volpe U., Fiorillo A. (2020). Validation of the brief TEMPS-M temperament questionnaire in a clinical Italian sample of bipolar and cyclothymic patients. J. Affect. Disord..

[59] Patel V., Burns J.K., Dhingra M., Tarver L., Kohrt B.A., Lund C. (2018). Income inequality and depression: A systematic review and meta-analysis of the association and a scoping review of mechanisms. World Psychiatry.

[60] Erbuto D., Innamorati M., Lamis D.A., Berardelli I., Forte A., De Pisa E., Migliorati M., Serafini G., Gonda X., Rihmer Z. (2018). Mediators in the association between affective temperaments and suicide risk among psychiatric inpatients. Psychiatry.

[61] Widiger T.A., Crego C. (2019). The five factor model of personality structure: An update. World Psychiatry.

[62] Batty G.D., Gale C.R., Tanji F., Gunnell D., Kivimäki M., Tsuji I., Jokela M. (2018). Personality traits and risk of suicide mortality: Findings from a multi-cohort study in the general population. World Psychiatry.

[63] Cuijpers P., Karyotaki E., Reijnders M., Purgato M., Barbui C. (2018). Psychotherapies for depression in low- and middle-income countries: A meta-analysis. World Psychiatry.

[64] Min J.A., Lee C.U., Lee C. (2013). Mental health promotion and illness prevention: A challenge for psychiatrists. Psychiatry Investig..

[65] Fisher J. (2020). Gender competence and mental health promotion. World Psychiatry.

[66] Ormel J., Cuijpers P., Jorm A.F., Schoevers R. (2019). Prevention of depression will only succeed when it is structurally embedded and targets big determinants. World Psychiatry.

[67] Campos R.C., Holden R.R., Costa F., Oliveira A.R., Abreu M., Fresca N. (2017). The moderating effect of gender on the relationship between coping and suicide risk in a Portuguese community sample of adults. J. Ment. Health.

[68] Blacker C.J., Lewis C.P., Swintak C.C., Bostwick J.M., Rackley S.J. (2019). Medical Student Suicide Rates: A Systematic Review of the Historical and International Literature. Acad. Med..

[69] Masten A.S. (2019). Resilience from a developmental systems perspective. World Psychiatry.

[70] Center C., Davis M., Detre T., Ford D.E., Hansbrough W., Hendin H., Laszlo J., Litts D.A., Mann J., Mansky P.A. (2003). Confronting depression and suicide in physicians: A Consensus Statement. J. Am. Med. Assoc..

[71] Rosenthal J.M., Okie S. (2005). White coat, mood indigo—Depression in medical school. N. Engl. J. Med..

[72] Dawson K.S., Watts S., Carswell K., Shehadeh M.H., Jordans M.J.D., Bryant R.A., Miller K.E., Malik A., Brown F.L., Servili C. (2019). Improving access to evidence-based interventions for young adolescents: Early Adolescent Skills for Emotions (EASE). World Psychiatry.

[73] Sinha M., Collins P., Herrman H. (2019). Collective action for young people’s mental health: The cities RISE experience. World Psychiatry.

[74] Pinna F., Del Vecchio V., Luciano M., Sampogna G., De Rosa C., Ferrari S., Luca P., Ilaria T., Umberto V., Carrà G. (2015). Shall psychiatry change its target? Reflections on the evolving role of psychiatry. Riv. Psichiatr..

[75] Fiorillo A., Malik A., Luciano M., Del Vecchio V., Sampogna G., Del Gaudio L., Kuzman M.R., Jovanovic N., Nawka A., Volpe U. (2013). Challenges for trainees in psychiatry and early career psychiatrists. Int. Rev. Psychiatry.

[76] Fiorillo A., Luciano M., Del Vecchio V., Sampogna G., Obradors-Tarragó C., Maj M., ROAMER Consortium (2013). Priorities for mental health research in Europe: A survey among national stakeholders’ associations within the ROAMER project. World Psychiatry.

[77] Pompili M., Fiorillo A. (2017). Editorial: Unmet Needs in Modern Psychiatry. CNS Neurol. Disord..

[78] Pinto da Costa M., Dima K., Ng R.M.K. (2019). Undergraduate psychiatric education: A snapshot of medical students across the world. World Psychiatry.

[79] Whitley R., Shepherd G., Slade M. (2019). Recovery colleges as a mental health innovation. World Psychiatry.

[80] Hanna F., Barbui C., Dua T., Lora A., van Regteren Altena M., Saxena S. (2018). Global mental health: How are we doing?. World Psychiatry.

